# Temporal patterns of fucoxanthin in four species of European marine brown macroalgae

**DOI:** 10.1038/s41598-023-47274-7

**Published:** 2023-12-14

**Authors:** Eoghan M. Cunningham, Aaron P. O’Kane, Lauren Ford, Gary N. Sheldrake, Ross N. Cuthbert, Jaimie T. A. Dick, Christine A. Maggs, Pamela J. Walsh

**Affiliations:** 1 School of Mechanical and Aerospace Engineering, Queen’s University Belfast, Belfast, BT9 5GA Northern Ireland, UK; 2 Queen’s University Marine Laboratory, Queen’s University Belfast, 12-13 The Strand, Portaferry, BT22 1PF Northern Ireland, UK; 3 School of Chemistry and Chemical Engineering, Queen’s University Belfast, Belfast, BT9 5GA Northern Ireland, UK; 4 Department of Metabolism, Digestion and Reproduction, Imperial College, London, UK; 5 Institute for Global Food Security, School of Biological Sciences, Queen’s University Belfast, 19 Chlorine Gardens, Belfast, BT9 5DL Northern Ireland, UK

**Keywords:** Marine biology, Marine chemistry

## Abstract

Brown seaweeds are a rich source of carotenoids, particularly fucoxanthin, which has a wide range of potential health applications. Fucoxanthin fluctuates within and among seaweeds over time, frustrating efforts to utilise this resource. Thus, we require comprehensive analyses of long- and short-term concentrations across species in field conditions. Here, we used High Performance Liquid Chromatography to compare fucoxanthin content in four brown macroalgae, *Ascophyllum nodosum, Fucus serratus*,* Fucus vesiculosus* and *Saccharina latissima*, monthly for 1 year. *F. serratus* and *F. vesiculosus* had significantly higher fucoxanthin content (mg/g), which was highest in Spring (0.39 ± 0.04) and Autumn (0.45 ± 0.04) [mean (± SE)]. Two species, *A. nodosum* and *F. serratus*, were collected monthly at the same location for a further two non-consecutive years. For both *A. nodosum* and *F. serratus*, a significant interaction effect of seasons and years was identified, highlighting that there is variation in fucoxanthin content among and within species over time. We also show that fucoxanthin content differs significantly among months even within seasons. Therefore, it is not sufficient to assess fucoxanthin in single months to represent seasonality. We discuss how weather, nutrients and reproduction may have driven the seasonal variation, and reveal patterns of fucoxanthin concentration that can provide information concerning its availability for many important medical functions.

## Introduction

**Figure 1:**
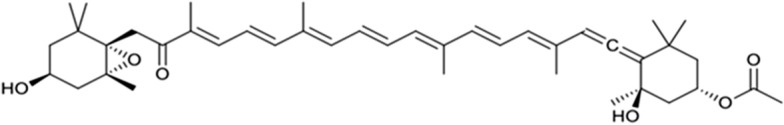
Fucoxanthin structure (drawn by AOK author in ChemDraw).

Brown macroalgae (Phaeophyceae, Ochrophyta) are a rich source of carotenoids, a class of naturally occurring yellow and orange pigments^1^, which comprise carotenes (such as β-carotene) and xanthophylls (e.g., canthaxanthin, astaxanthin, zeaxanthin and fucoxanthin^2^). β-carotene functions as an antenna pigment providing photooxidative protection against UV-B light^3^ and is a precursor of vitamin A which is required for growth and tissue repair^4^, whereas xanthophylls are carotenoids that are involved in photosynthesis and are found in the peripheral antenna complexes, functioning as efficient accessory light-harvesting pigments^5^ which transfer energy to the photosystems^6^. In brown seaweeds, xanthophylls are produced in large quantities after exposure to specific environmental stimuli such as radiation^7^. Fucoxanthin (Fig. 1) is found only in brown macroalgae and diatoms, representing 10% of all their carotenoids^8^, and plays essential roles in light harvesting and antioxidant functions^5,9^. It consists of two functionalised cyclohexane rings linked by a long, polyene chain. The functional groups include two free hydroxyl groups, one on each ring, an acetylated hydroxyl group, an epoxide and an unusual allene at one end of the polyene chain. A carbonyl group is present at the other end of the polyene. The polyene chain acts as a chromophore with the compound being a bright yellow colour. It is a more efficient accessory light-harvesting pigment than other carotenoids with a similar light absorption region^10,11^. Fucoxanthin may also have a photoprotective role in brown algae like other carotenoids in flowering plants^12^.

Fucoxanthin has a wide range of bioactivities with potential applications in human health^13,14^, having anti-obesity^15^, anti-inflammatory^16,17^ and anti-cancer effects^18,19^. It has been shown to reduce plasma and hepatic lipid profiles^20^ and to reduce photodamage through ultraviolet B radiation when applied to skin^13^. It is an antioxidant, based on its radical scavenging ability^21^, an antidiabetic, evidenced by improved insulin resistance^22^, and has antimalarial^13^ and antibacterial effects^23^.

The global market for fucoxanthin is currently USD 31 million, with 3.9% predicted growth over the next 6 years^24^. Its commercial translation has been dominated by the health supplements market, e.g., Xanthigen® and FucoVital™, although interest is also growing commercially in its application as a natural, eco-friendly colourant^25^.

Fucoxanthin has been extracted from a wide range of brown seaweeds including the subtidal kelps *Saccharina japonica* and *Undaria pinnatifida* and intertidal fucoids, e.g., *Fucus serratus, Fucus vesiculosus* and *Ascophyllum nodosum*^13^. The fucoxanthin content of macroalgal species is reported to vary according to various biotic and abiotic factors. In *A. nodosum*, a complex pattern of variation in pigment content was found in relation to tissue age, exposure to light, and nutrient conditions. For example, tips less exposed to light and older tissue, which is naturally shaded, had higher fucoxanthin than the light-exposed tips^26^. However, contrasting results have been found more recently, and there is no consensus as yet on the effects of light and tissue age on fucoxanthin content in fucoids^27^. Increased fucoxanthin levels have been found in the sugar kelp *Saccharina latissima* in relation to decreased temperature and irradiance^28,29^, greater water depth^28^, and increased nutrient levels in aquaculture settings^30,31^. Fucoxanthin content increases as kelp sporophytes mature^32–34^.

Seasonality is also known to affect fucoxanthin content in macroalgae. To date, relatively few studies have considered the impact of seasonal variation on fucoxanthin yields across a range of species. In Northern Ireland, pigment (chlorophyll *a* and fucoxanthin) levels in tips of *A. nodosum* were highest in winter (January) and lowest in summer (July)^26^. Similar results were found on the west coast of Ireland (high in February, low in June)^35^. In *F. serratus*, fucoxanthin showed a similar pattern of low values in June and October, and high levels in February^35^. Likewise, the fucoxanthin content of *Sargassum horneri* (Sargassaceae) in Japan was maximal in winter (January) and then decreased during subsequent months^36^. In contrast, kelps seem to show a reversed pattern of fucoxanthin abundance. In *S. latissima*, the lowest values were in February, and highest in October^35^. This correlated with the decrease in fucoxanthin in *S. latissima* at greater depths in Portugal^31^. However, the opposite was found in Norway: high temperatures (15 °C and 20 °C) resulted in a significant decrease in both chlorophyll *a* and fucoxanthin. Two European studies^30,31^ have shown that nutrient status has a major effect in *S. latissima*, with four-fold higher fucoxanthin contents in seaweeds grown in high-nutrient Integrated Multi-Trophic Aquaculture systems.

It is clear that there is as yet no consensus on whether fucoxanthin levels are higher in winter or summer, or whether fucoids and kelps are similar or show contrasting patterns. At present, the seasonality of pigment content in brown macroalgae is poorly understood. One common concern is the use of a broad seasonal scale (e.g., summer, winter) for sampling, potentially confusing the pattern. Given the importance of fucoxanthin for applications in pharmaceutical and functional food industries^14,37^, a dependable year-round source needs to be identified to maximise use of such a commodity. To establish a dependable source, further investigation of the contrasting reports of fucoxanthin content in various brown seaweed species, particularly across seasons, is required. Hence, in this study, we examined fucoxanthin content in four macroalgal species abundant on European Atlantic shores, the fucoids *A. nodosum, F. vesiculosus* and *F. serratus*, and the kelp *S. latissima*. The species were sampled monthly on the northeast coast of Ireland over one year, and for two of these species, *A. nodosum* and *F. serratus*, two further years of monthly sampling were undertaken. The aim of this study was to determine how fucoxanthin content differed in relation to species, months, seasons, and across multiple sample years.

## Materials and methods

### Collection site and sampling

**Figure 2:**
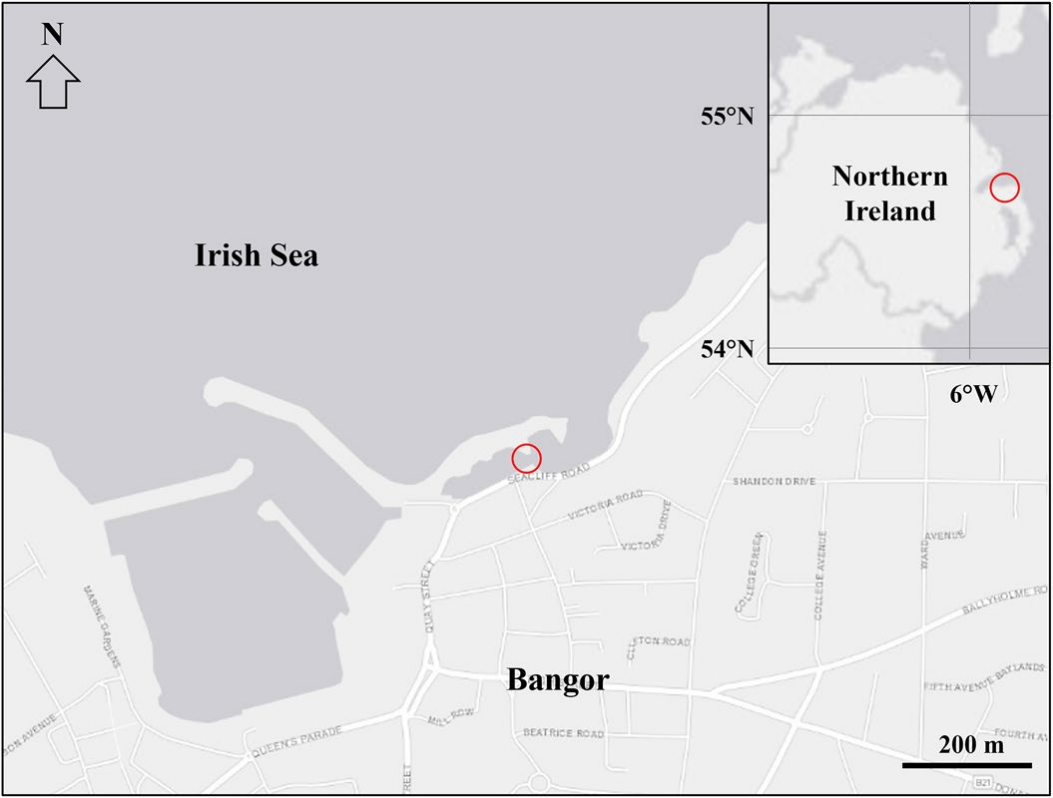
Collection Site at Bangor, Co. Down, Northern Ireland (54° 39′ 58.6″ N 5° 39′ 53.4″ W).

Fucoxanthin content was studied in fucoid and kelp species growing in an artificial lagoon behind a breakwater at Bangor, Co. Down, Northern Ireland (54° 39′ 58.6″ N 5° 39′ 53.4″ W; Fig. 2). This location was chosen as it is a sheltered growth platform providing convenient access to the intertidal and upper sublittoral zones over a 2.2 m tidal range. The site is adjacent to various recreational facilities (e.g., large yacht marina, sailing club and promenade), and is of low conservation interest with no marine protection designations.

**Figure 3:**
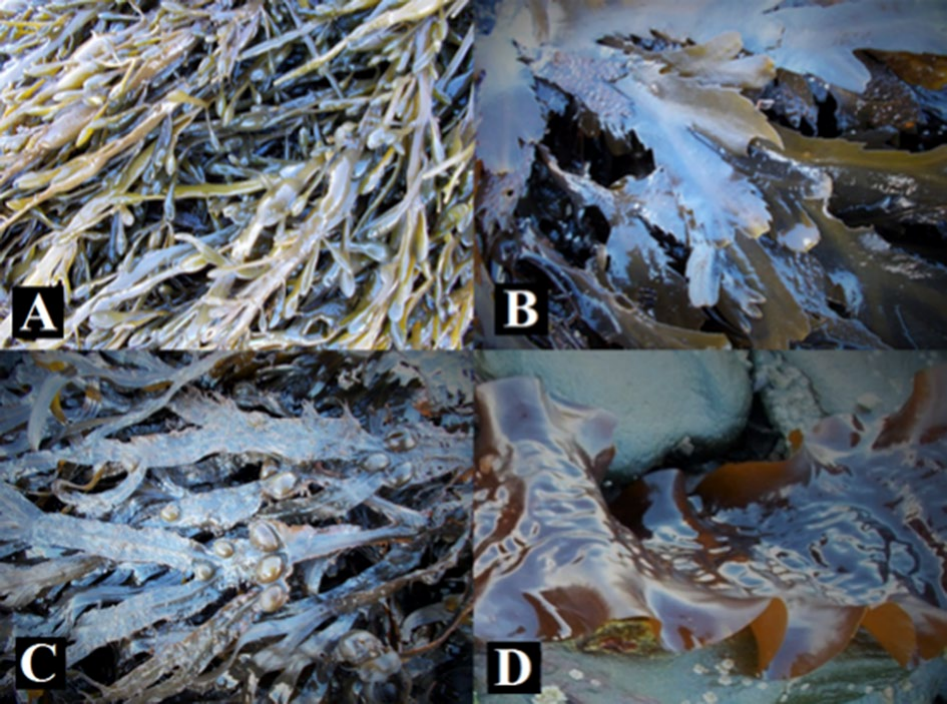
Macroalgal species studied: (**A**) *A. nodosum*, (**B**) *F. serratus*, (**C**) *F. vesiculosus*, (**D**) *S. latissima*.

The collection site had a diverse range of macroalgal species that could be sustainably harvested for this study without a significant impact on the communities. The four most abundant brown macroalgal species were selected for this study: *A. nodosum* (L.) Le Jolis*, F. serratus* L., *F. vesiculosus* L., and S. latissima (L.) C.E. Lane, C. Mayes, Druehl & G.W. Saunders (Fig. 3).

On a monthly basis, ~ 1 kg wet weight of multiple individuals of each fucoid species and for the kelp (blades only) were collected haphazardly in the littoral zone (fucoids) and upper sublittoral zone (kelp) of our study site. The total wet weight per species was then subsampled into three roughly equal replicates by weight to give three replicates for each species and time point. For year one (October 2015–September 2016), all four species were sampled monthly and replicated three times as above (i.e., 4 species × 12 months × 3 replicates = 144 samples). In addition to year one, *A. nodosum* and *F. serratus* were sampled in the same manner for year two (January 2018–December 2018) and year three (January 2021–December 2021) (i.e., each year = 2 species × 12 months × 3 replicates = 72 samples).

### Sample preparation and fucoxanthin analysis

After collection, each macroalgal subsample was roughly cut into small pieces of approx. 1–2 cm with scissors, blotted dry, and placed in a 500 mL round-bottomed flask. The flask was then frozen overnight at − 20 °C, and water was removed over 2 days using a Savant Modulyo freeze dryer with an Edwards RV8 vacuum pump. To ensure a homogeneous particle size, the samples were then ground using a Polymix PX-MFC 90 D grinder and passed through a 2 mm sieve. Samples were stored in a freezer at − 20 °C in a plastic sample bag until further use. For analysis, three technical replicates were taken from each biological replicate of ground material for each extraction.

Extraction of fucoxanthin was performed using a modification of Fung et al.'s method^33^. 1 g dry weight per extraction of dried macroalgal samples was stirred in 20 mL methanol (Sigma-Aldrich UK, anhydrous, 99.8%; 20:1 V:W) for 1 h at room temperature and left to settle overnight to aid filtration. All extractions were performed away from direct sunlight and samples were stored in darkness overnight. All three replicate samples were extracted and analysed individually to improve accuracy. The supernatant was filtered through cotton wool and analysed immediately using High Performance Liquid Chromatography (HPLC; Agilent 110 series HPLC system). Fucoxanthin was separated on a Supelco Ascentis C18 column, 250 mm × 4.6 mm with 5 μm stationary phase. The mobile phase was 100% methanol with a flow rate of 1 mL min^−1^ with sample injection volume of 10 μL at a column temperature of 28 °C. The absorbances were measured at a wavelength of 450 nm on the UV–Vis detector. The standard curve (Fig. S3) and retention times were calibrated using a pure fucoxanthin standard (Sigma-Aldrich UK; Product Code: 1001590265; Lot No: #SLBG8952V) whereby 10 mg of the standard was dissolved in 100 mL methanol.

### Statistical analysis

All statistical analyses were computed in R v3.4.4^38^, visualised using ggplot2 v3.4.1^39^, and juxtaposed using cowplot v1.1.1^40^. Gamma generalized linear mixed models (GLMMs) fitted with a logarithmic link function were determined to have the best model fit using LMERConvenienceFunctions v3.0^41^. Four different models were created for the analysis using the function *glmer* in lme4 v1.1–27.1^42^. For seasonal analyses, the meteorological seasons were used, i.e., winter = December, January, February; spring = March, April, May; summer = June, July, August; autumn = September, October, November. For the year one data consisting of all four species, two separate models were created to avoid the temporal correlation of months and seasons: These two models assessed fucoxanthin content with respect to (1) ‘species’ and ‘season’ and their interactions, and separately, (2) ‘species’ and ‘months’ and their interactions. For the multi-year data, the fucoxanthin content was assessed in relation to ‘seasons’ and ‘years’ and their interaction in two separate models for the two species: (1) *A. nodosum* and (2) *F. serratus*. The species were modelled separately as we were interested in how the fucoxanthin content of each individual species varied over time. For each of the mixed models, ‘replicate’ was added as a random intercept to determine if the fixed effects were influenced by subsample differences. For model (2), ‘season’ was added as an additional random intercept to account for months within seasons. Type III sum of squares analyses of deviance for each variable were performed using the *Anova* function within car v3.0-10^43^. Tukey's comparisons based on the GLMMs were used post hoc for pairwise comparisons of significant effects using emmeans v1.8.5^44^.

## Results

### Year one analysis: four species

**Figure 4:**
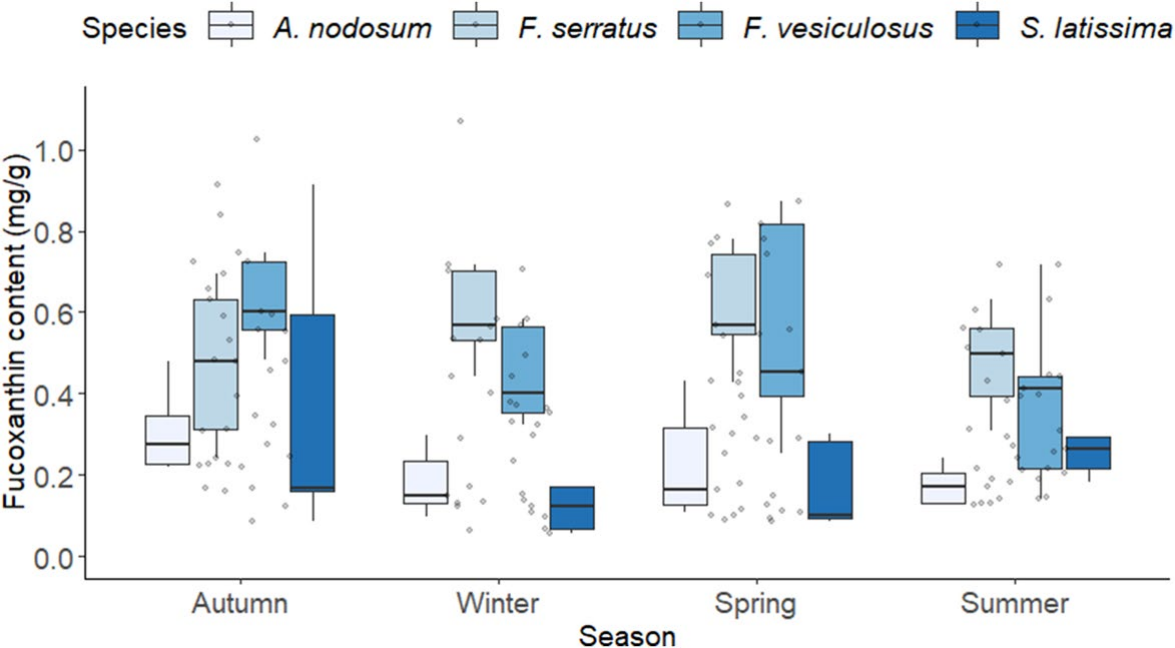
Fucoxanthin content of the four macroalgae (*A. nodosum*, *F. serratus*, *F. vesiculosus*, *S. latissima*) during four seasons (Autumn, Winter, Spring, Summer). All data were collected during year one (Oct 2015–Sep 2016). Each box and whisker plot shows the median, inter-quartile range, and min/max values. The raw data are shown as points on each plot (*n* = 9 per species per season).

When looking at individual species and seasons in year one, consistently low levels of fucoxanthin were found in *A. nodosum*, with low but variable levels identified in *S. latissima*. Comparativley, the two *Fucus sp.* had much higher values, with some evidence of seasonaility driving fucoxanthin content, i.e., highest values found in Autumn (0.45 ± 0.04 mg/g) and lowest values in summer (0.33 ± 0.03; Fig. 4; Table S1). In addition, a significant species:seasons interaction effect was found (χ^2^_1,9_ = 29.78, *P* < 0.001), highlighting that the differences among the four species were mediated by seasonality. The fucoxanthin contents of the two *Fucus* species did not differ significantly during any season. Both species consistently displayed higher fucoxanthin contents than the other fucoid and kelp species in all four seasons (Fig. 4; Table S1). Fucoxanthin content of *F. serratus* was highest in Spring (max 0.78 mg/mg) and lowest in Autumn (min 0.24), while *F. vesiculosus* had the highest values in Winter (max 1.07) with the lowest found in Summer (min 0.14). Interestingly, while Summer showed the lowest fucoxanthin contents overall, the values identified in *F. serratus* were still significantly higher than in other species during this time (Fig. 4; Table S1). *A. nodosum* and *S. latissima* consistently displayed lower fucoxanthin contents than the two *Fucus* species, *A. nodosum* was found to be highest in Autumn (max 0.48) and lowest in Winter (min 0.09) and *S. latissima* was found to be highest in Autumn (max 0.91) and lowest in Winter overall (min 0.05; Fig. 4). In addition, Summer was the only season where *S. latissima* showed a significantly greater fucoxanthin content than another species (*A. nodosum*; see Table S1 for all pairwise differences).

**Figure 5:**
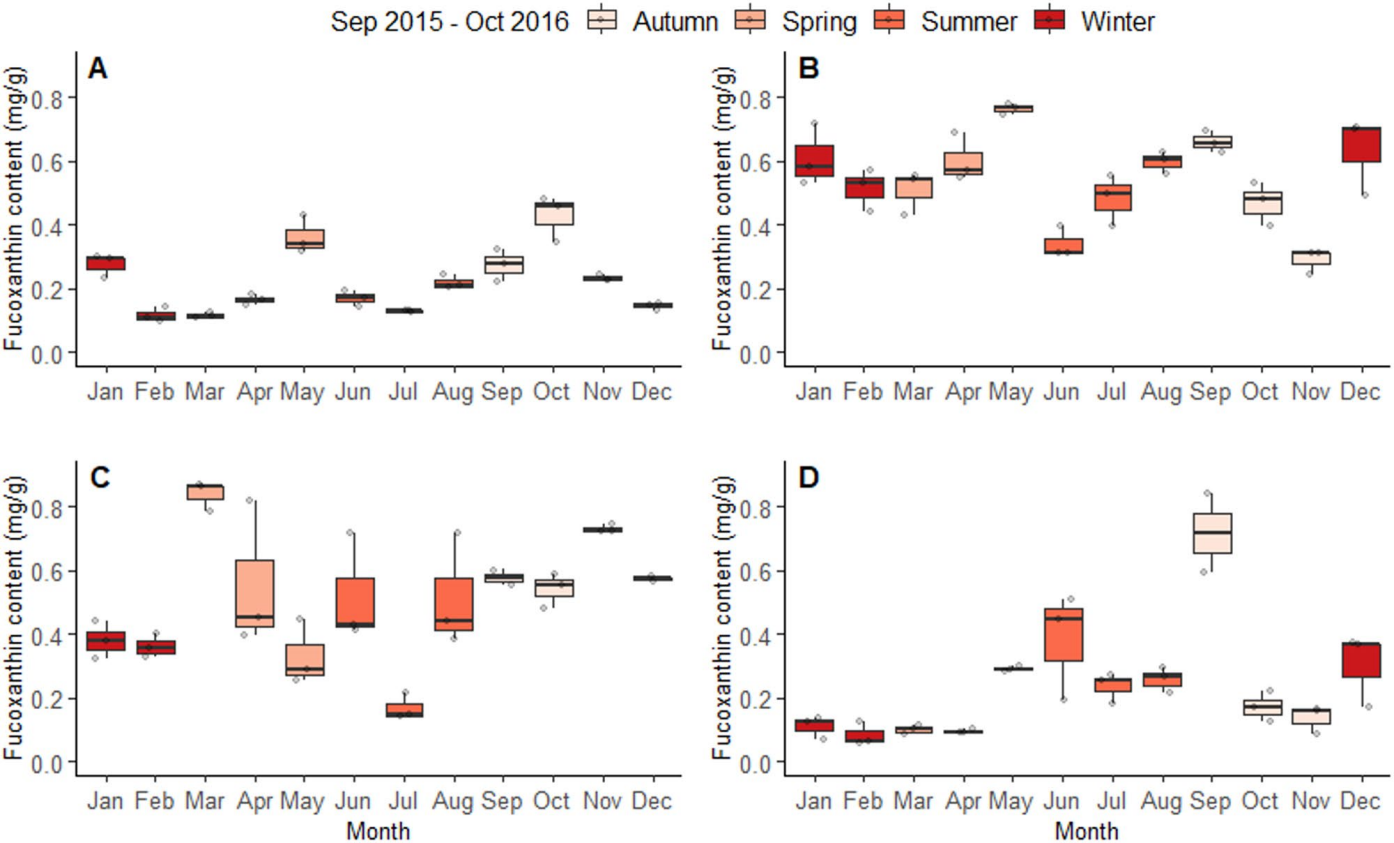
The fucoxanthin content of *Ascophyllum nodosum* (**A**), *F. serratus* (**B**), *F. vesiculosus* (**C**), and *S. latissima* (**D**), over each of the 12 months within year one. Each box and whisker plot shows the median, inter-quartile ranges, and min/max values. The raw data are shown as points on each plot (*n* = 3 per month).

The significant seasonal differences identified in the previous paragraph were also underpinned by monthly fucoxanthin differences. The fucoxanthin content of each species showed signficant monthly differences within seasons, which was highlighted by the second model through a significant species:months interaction effect (χ^2^_1,33_ = 632.87, *P* < 0.001). In *A. nodosum*, significant monthly differences in fucoxanthin content were found within all seasons (Fig. 5A; Table S2). No significant differences were found in the fucoxanthin content of *F. serratus* among the Winter and Spring months, while Summer and Autumn all had significant monthly differences (Fig. 5B; Table S2). In *F. vesiculosus*, fucoxanthin content was shown to differ significantly within the months of Winter, Spring, and Summer, with no differences being found among Autumn months (Fig. 5C; Table S2). Finally, significant monthly differences in fucoxanthin content were found within all seasons in *S. latissima* (Fig. 5D; Table S2).

### Multi-year analysis: two species

**Figure 6:**
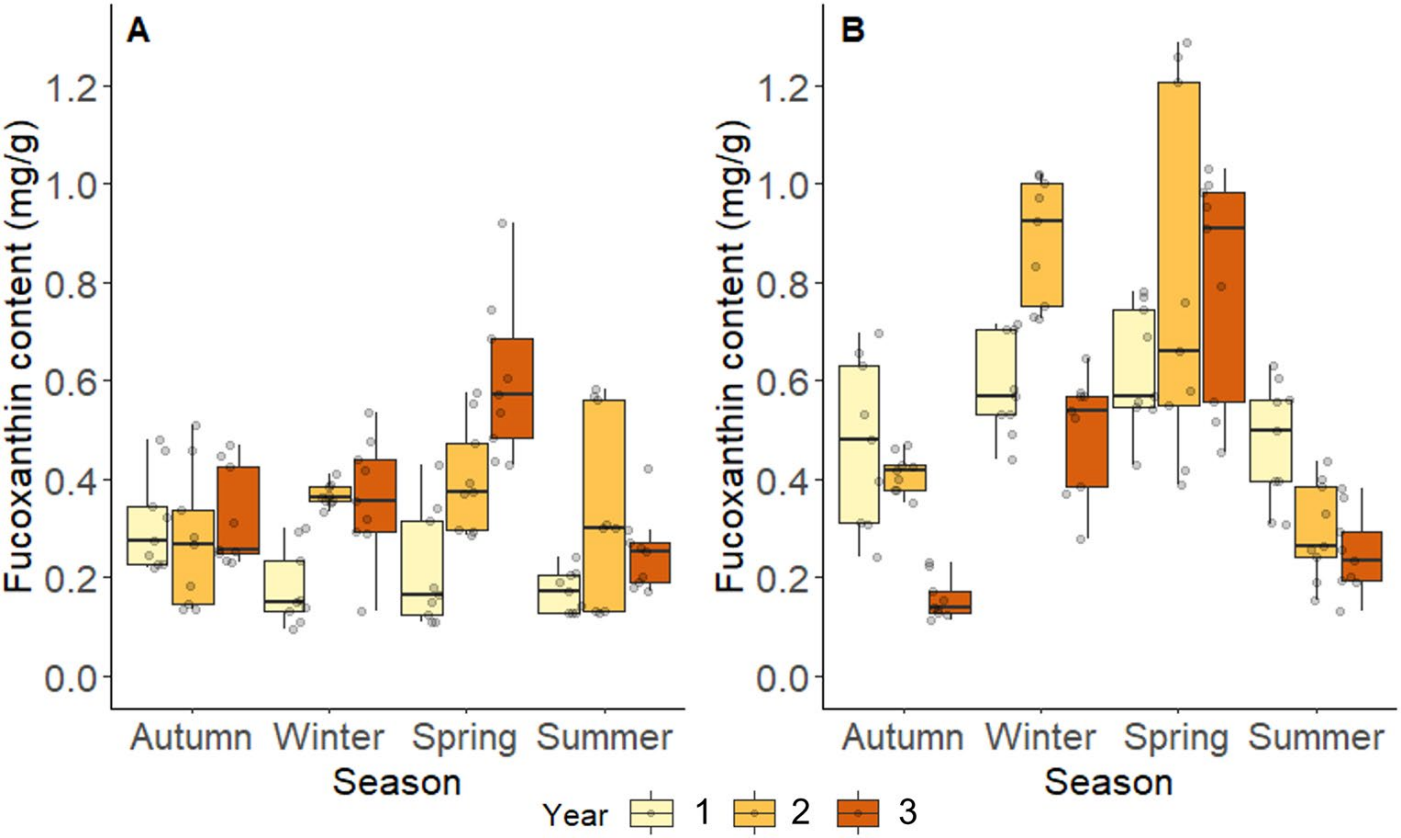
The fucoxanthin content measured in *A. nodosum* (**A**) and *F. serratus* (**B**) across four seasons (Autumn, Winter, Spring, Summer), and three years (year one; Oct 2015–Sep 2016, year two; Jan 2018–Dec 2018, year three; Jan 2021–Dec 2021). Each box and whisker plot shows the median, inter-quartile ranges, and min/max values. The raw data are shown as points on each plot (*n* = 18 per season) .

Across years two and three, fucoxanthin content was highest overall in *F. serratus*, which displayed annual mean (± SE) values of 0.59 ± 0.05 and 0.42 ± 0.04, respectively (Fig. 6B). In comparison, the fucoxanthin contents of *A. nodosum* for years two and three were 0.34 ± 0.02 and 0.38 ± 0.03 (Fig. 6A). A significant interaction effect between years and seasons was also found in the multi-year *A. nodosum* data (χ^2^_1,6_ = 31.74, *P* < 0.001). This effect was highlighted by significantly lower fucoxanthin content identified in Summer in comparison to Autumn (Z = 3.58, *P* = 0.002) and Winter (Z = 3.36, *P* = 0.004) in year one. By contrast, in year two, no seasonal differences in fucoxanthin content were found. However, in year three, Spring showed significantly higher values than Autumn (Z = − 3.80, *P* < 0.001), Summer (Z = 5.29, *P* < 0.001), and Winter (Z = 3.06, *P* = 0.012).

In addition, a significant interaction effect between years and seasons was also found for *F. serratus* (χ^2^_1,6_ = 84.87, *P* < 0.001), which was driven by significant seasonal differences in year two and three. During year two, fucoxanthin content was significantly higher in Spring than Autumn (Z = − 5.11, *P* < 0.001) and Summer (Z = 7.77, *P* < 0.001). Winter also showed significantly higher values than both Autumn (Z = − 6.02, *P* < 0.001) and Summer (Z = − 8.67, *P* < 0.001). In year three, significant seasonal differences were found among all seasons, with Spring and Winter both reporting the highest values (Fig. 6B).

**Figure 7:**
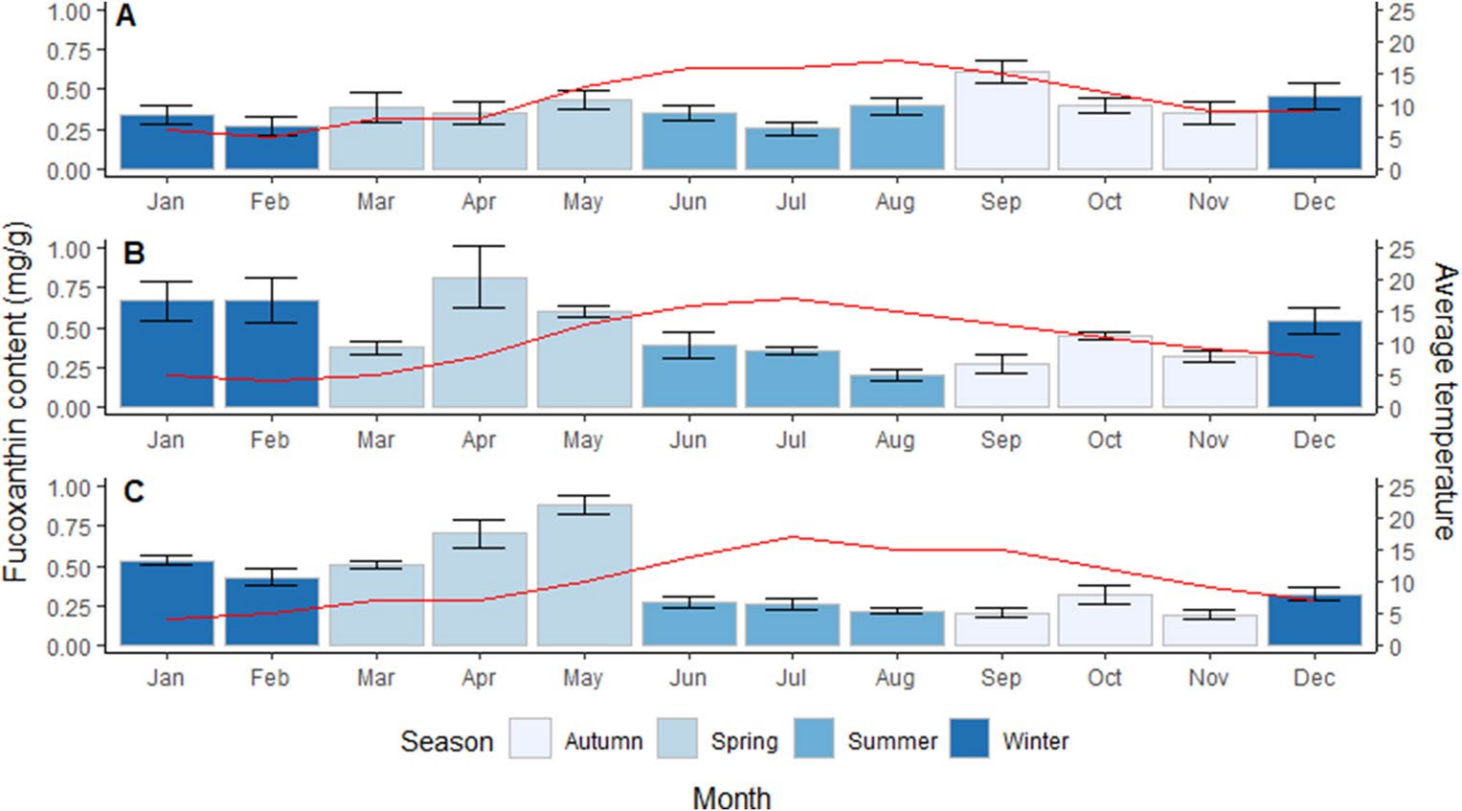
The monthly and seasonal fucoxanthin content measured across all species sampled in year one (**A**), year two (**B**), and year three (**C**). The mean (± SE) fucoxanthin content is displayed for each month as barplots with error bars. Average monthly temperature measured at the study site is displayed as a red line on each plot. Temperature data from Bangor, Northern Ireland sourced from Met Office UK.

For each of the three sampled years, there was also a clear trend in the fucoxanthin content in the study species in relation to the average monthly temperature at the study site (Fig. 7). Across all three years, the fucoxanthin content was consistently lowest in the Summer months when the monthly average temperatures were at their highest. In year one, the fucoxanthin contents were higher in Spring and Autumn before and after the average monthly temperatures peaked. However, in the second and third year, fucoxanthin content was highest in Spring and Winter, appearing to increase with temperature ahead of the summer temperature peak.

## Discussion

### Species differences

*Fucus serratus, F. vesiculosus* and *A. nodosum* are intertidal species whereas *S. latissima* is usually found in the low intertidal and shallow subtidal. Seaweeds typically found at higher elevations in the littoral zone tend to be more tolerant to environmental fluctuations, whereas those at the lowermost intertidal zone (with little or no emersion at low tide) are less tolerant of these environmental stresses^45^. In this study, species were collected randomly within the littoral and upper sublittoral zones at one site, to reflect commercial harvest practice, and to help gain an understanding of the resultant variation in fucoxanthin content in seaweed biomass collected in this manner. Direct comparison between species is difficult due to intra- and interspecific factors (e.g., genetic differences, thallus morphology) and also environmental factors between collection sites (e.g., water depth, nutrient availability and self-shading) that are known to influence the pigment profile of brown seaweeds^25,26^. For example, in kelps different thallus morphological profiles have been shown to cause significant variation in photosynthetic pigments^46–48^. Regardless, similar trends were observed in this study to those reported by other studies. In a recent study by Rubino et al.^25^ 12 different species of brown seaweeds were screened (including Fucaceae and kelp), with fucoxanthin content found to be highest in *F. serratus* followed by *F. vesiculosus*^25^*.* An earlier study from Ireland also reported a higher fucoxanthin content in *F. vesiculosus* compared to *A. nodosum* collected from the same site^49^, which also corroborates our study. However, an Icelandic study reported the opposite trend to Rubino et al.^25^ whereby *A. nodosum* was found to have a higher content than *F. vesiculosus* which they attributed to light and air exposure of the seaweeds at the collection site^50^. There have been few direct comparisons of the fucoxanthin content between species using the whole fronds (as per this study) as opposed to sections (e.g., tips of blades) of the seaweed. This makes it difficult to compare our results with those reported in the literature.

### Seasonality

Our results support the findings from several other studies on different brown seaweeds^26,31,35,51,52^, that reported lower fucoxanthin content in Summer. However, Heffernan et al. reported the opposite trend with the highest content of fucoxanthin observed in summer months for *F. serratus*^37^*.* It is not clear from their methodology if samples were collected at monthly intervals to represent seasonality or if they only collected samples in one month to represent seasonal trends. During the summer months, all species are exposed to more desiccation stress due to higher average air temperatures (Fig. S2), longer daylight hours and more solar irradiance when compared to other seasons. Although sunshine does not necessarily equal photon irradiance (as sun in the autumn could equal a dull summer), seaweeds are exposed to much more prolonged periods of high photon irradiance in the summer than in winter. Solar irradiance increases from c. 20 W m^−2^ in Dec/Jan to almost 200 W m^−2^ in July in the UK^53^. The overall lower fucoxanthin content in all four species in summer is most likely therefore due to high solar irradiance exposure during summer months, and longer daylight hours that can also potentially coincide with longer exposure to air (during summer low tides). Macroalgae are known to reduce the amount of accessory pigments to avoid excessive light capture^54^, to prevent damage to their photosystems^55^. Although seaweeds are not exposed throughout the tidal cycle, the intensity of the light exposure is much greater in summer, which is likely to contribute to lower fucoxanthin contents being found at this time. Sampath-Wiley et al.^45^ reported that sun exposure had more of an effect on the photosynthetic pigments than desiccation in red seaweeds. However, further work is required to determine which factors, intensity of solar irradiance, dehydration/rehydration of seaweeds, or a combination of both, has a greater impact on pigmentation, specifically fucoxanthin.

### Sampling monthly versus seasonality

Although our seasonal results differ from earlier studies mentioned, those studies, e.g., Zacharias^35^ (*S. latissima*, Ireland) and Stengel and Dring^26^ (*A. nodosum* tips, N. Ireland) only sampled one month per season. Our data show that there were monthly significant differences within seasons across all four of our study species (Table S2). Moreover, for the two species measured inter-annually, there were significant season effects over multiple years even at a single site, further indicating the challenges of reliance on temporal snapshots or short-term studies in the field. These differences highlight that representative seasonal sampling for fucoxanthin content cannot be carried out using only one month per season, therefore, we recommend that future studies should sample all months within the season to ensure greater comparability among studies. However, while monthly differences did occur within seasons, the drivers behind these changes are currently unknown, and we are unsure if these changes will make a difference for industrial sampling techniques where multiple species are collected and processed as part of one crude/mixed sample.

### Nutrient levels

It is also noteworthy that our study site is within an agricultural catchment area and is seasonally affected by eutrophication, which has also shown to influence the fucoxanthin content in brown seaweeds^26^. Seasonal differences in fucoxanthin content may also be impacted by nutrient levels, such as the reduced nutrient levels found typically during summer months in temperate marine systems. However, increases in fucoxanthin content in *S. latissima* have been correlated to increased nutrient levels in aquaculture systems in the past^30,31^.

### Reproduction

The formation of receptacles (reproductive structures), and indeed, the sex of the fucoid species studied here could also help to explain the seasonal variation in fucoxanthin. For example, it is characteristic of male *F. serratus* plants to display increased levels of the pigment carotene in their receptacles when they become fertile, which turns the structure an orange colour. A previous study investigating pigment concentrations in small amounts of thallus material from Baltic Sea *F. vesiculosus* found that fucoxanthin and carotene both differed in relation to the sex and reproductive status of the plant^56^. Fucoxanthin content was higher in vegetative plants, but even the reproductive females had higher levels in comparison to males. In contrast, carotenes were higher in the reproductive males. In our study, we saw a dip in the fucoxanthin content of *A. nodosum* during the winter months followed by a peak in May. This pattern corresponds with the reproductive cycle of *A. nodosum* at our study site and is therefore likely to be related to the formation of receptacles, followed by their subsequent shedding once gametes have been released. This highlights that industrial sampling of macroalgae for fucoxanthin content must consider the reproductive stages of the target species to avail of the higher concentrations found in vegetative plants, and to ensure that not all sporing bodies are removed to ensure harvesting be made more more sustainable.

### Grazing pressure

It is also likely that the seasonal differences in fucoxanthin content found here are also influenced by herbivorous grazers, such as molluscs and crustaceans. In the past, large reductions in the concentration of photosynthetic pigments have been caused by selective grazing pressure on photosynthetic tissue in the kelps *Lessonia spicata*^57^ and *Macrocystis pyrifera*^58^, but little information is available for the species studied here. At our study site, grazer activity peaks during the summer months, which may help to explain why summer had the lowest levels of fucoxanthin content among all our study species. It is also possible that the combinations of both abiotic and biotic stressors such as increased irradiance and grazing pressure are driving the reduction in fucoxanthin content in summer, however, the possible synergistic effects of multiple stressors require further investigation.

### Conclusion

The efficiency and predictability of fucoxanthin harvests from seaweeds is mediated by temporal variations both among and within species. The results of this study thus suggest that other factors that we did not investigate (e.g., weather, nutrient status, reproduction, and predation by grazers) which are tied to seasonality, may significantly influence fucoxanthin content. These factors may also have contributed to conflicting results in different literature studies. However, our information is useful for biotechnology industries to help determine which species would be best to harvest and the best time to harvest them to obtain fucoxanthin cost-effectively. For industry, as the commercial markets for these compounds continue to grow, the analysis of the whole seaweed biomass (i.e., the fronds) is more relevant than selective sampling to overall understanding of seasonal trends when collecting seaweed. Whilst seaweed harvesting practise has shifted in recent years, from full to partial removal (leaving holdfast) of the seaweed to aid recovery rates and recolonisation of collection sites, it is not feasible for industry to separate the tips from the fronds or other parts of the plant for a marginal improvement in yield. Therefore, studies which examine the overall trends in fucoxanthin content are more beneficial to industry, to help determine not only the best time of year to harvest, but also the best species. Our results suggest that in terms of fucoxanthin quantity within the seaweed biomass, *Fucus* species would be the richest. However, when making decisions on an industrial scale, other more complex factors need to be considered including the quantities of biomass harvested and the harvesting cost, costs of additional processes to recover fucoxanthin, the cost of implementing this process into existing technologies, and the environmental and ecological impact of harvesting. As the fucoxanthin contents found here are as high as any others reported, the results of this study have highlighted the value of, and the need for more research into these highly productive European seaweeds.

### Supplementary Information

Supplementary Information 1.

Supplementary Information 2.

## Data Availability

All data generated or analysed during this study are included in this published article [and its supplementary information files].
