# Supplementary material for: Temporal patterns of fucoxanthin in four species of European marine brown macroalgae
Source: Sci Rep. 2023 Dec 14;13:22241. doi: 10.1038/s41598-023-47274-7 (PMC10721839; doi:10.1038/s41598-023-47274-7)
Supplement: Supplementary file 1 — Supplementary Information 1. [file 41598_2023_47274_MOESM1_ESM.docx]

**Temporal patterns of fucoxanthin in four species of European marine brown macroalgae**

Eoghan M. Cunningham^1,2^, Aaron P. O’Kane^3^, Lauren Ford^4^, Gary Sheldrake^3^, Ross N. Cuthbert^2,5^, Jaimie T.A. Dick^2,5^, Christine A. Maggs^2^ & Pamela J. Walsh^1,2^*

**Supplementary material**


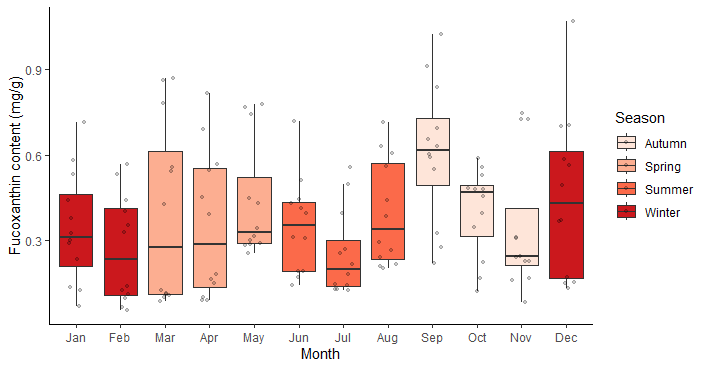


**Figure S1:** The combined fucoxanthin content of the four macroalgal species (*Ascophyllum, nodosum*, *Fucus serratus*, *F. vesiculosus*, and *Saccharina latissima*) identified during each month of sample year one. Raw data points include all four species for each month (*n* = 12 per month)


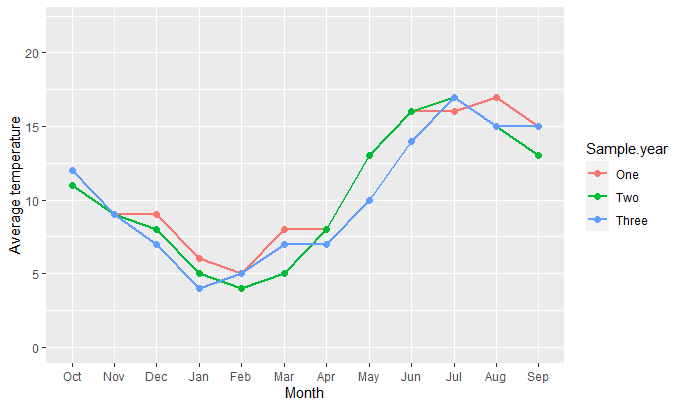


**Figure S2:** Monthly average temperature (°C) data for each month sampled during sample year one, two, and three. Source: Met Office UK, Bangor, Northern Ireland.


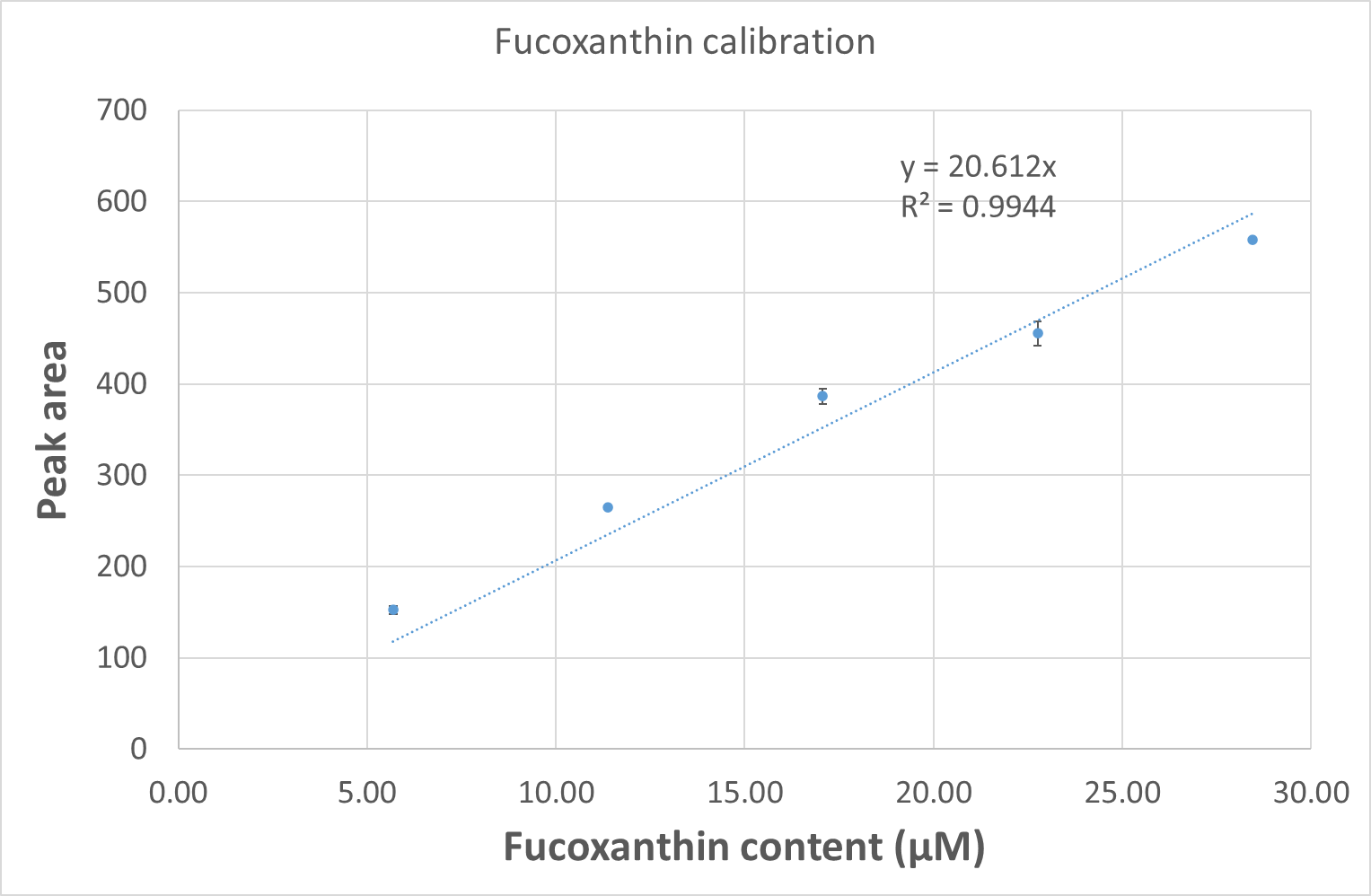


**Figure S3:** Calibration curve for pure Fucoxanthin (Sigma-Aldrich UK; Product Code: 1001590265; Lot No: #SLBG8952V)

**Table S1: P**airwise species differences in fucoxanthin content within each season (Autumn, Spring, Summer, Winter) for each of the four macroalgal species (*Ascophyllum, nodosum*, *Fucus serratus*, *F. vesiculosus*, and *Saccharina latissima*). Data include Season, Species, Z ratio (Tukey pairwise difference), and *P* value.

| **Season** | **Species** | **Z ratio** | ***P* value** |
| --- | --- | --- | --- |
| Autumn | *A. nodosum - F. serratus* | -2.05 | =0.168 |
| Autumn | *A. nodosum - F. vesiculosus* | -3.76 | =0.001 |
| Autumn | *A. nodosum - S. latissima* | -0.76 | =0.871 |
| Autumn | *F. serratus - F. vesiculosus* | -1.70 | =0.319 |
| Autumn | *F. serratus – S. latissima* | 1.29 | =0.567 |
| Autumn | *F. vesiculosus - S. latissima* | 3.00 | =0.014 |
| Spring | *A. nodosum - F. serratus* | -5.29 | <0.001 |
| Spring | *A. nodosum - F. vesiculosus* | -4.88 | <0.001 |
| Spring | *A. nodosum - S. latissima* | 1.38 | =0.507 |
| Spring | *F. serratus - F. vesiculosus* | 0.41 | =0.976 |
| Spring | *F. serratus – S. latissima* | 6.68 | <0.001 |
| Spring | *F. vesiculosus - S. latissima* | 6.27 | <0.001 |
| Summer | *A. nodosum - F. serratus* | -5.02 | <0.001 |
| Summer | *A. nodosum - F. vesiculosus* | -4.19 | <0.001 |
| Summer | *A. nodosum – S. latissima* | -2.63 | 0.042 |
| Summer | *F. serratus - F. vesiculosus* | 0.82 | =0.841 |
| Summer | *F. serratus – S. latissima* | 2.39 | =0.079 |
| Summer | *F. vesiculosus - S. latissima* | 1.56 | =0.399 |
| Winter | *A. nodosum - F. serratus* | -5.88 | <0.001 |
| Winter | *A. nodosum - F. vesiculosus* | -5.04 | <0.001 |
| Winter | *A. nodosum – S. latissima* | 0.39 | =0.978 |
| Winter | *F. serratus - F. vesiculosus* | 0.84 | =0.835 |
| Winter | *F. serratus – S. latissima* | 6.28 | <0.001 |
| Winter | *F. vesiculosus - S. latissima* | 5.44 | <0.001 |

**Table S2:** Pairwise differences of fucoxanthin content between months within seasons (Autumn, Spring, Summer, Winter) for each of the four macroalgal species (*A. nodosum*, *F. serratus*, *F. vesiculosus*, and *S. latissima*). Data include Species, Season, Months, Z ratio (Tukey pairwise difference), and *P* value.

| **Species** | **Season** | **Months** | **Z ratio** | ***P* value** |
| --- | --- | --- | --- | --- |
| *A. nodosum* | Autumn | Sep - Oct | -8.74 | <0.001 |
| *A. nodosum* | Autumn | Oct - Nov | -11.48 | <0.001 |
| *A. nodosum* | Autumn | Nov - Sep | -2.74 | =0.206 |
| *A. nodosum* | Spring | Mar - Apr | 6.77 | <0.001 |
| *A. nodosum* | Spring | Mar - May | -21.92 | <0.001 |
| *A. nodosum* | Spring | Apr - May | -15.15 | <0.001 |
| *A. nodosum* | Summer | Jun - Jul | -5.00 | <0.001 |
| *A. nodosum* | Summer | Jul - Aug | 10.16 | <0.001 |
| *A. nodosum* | Summer | Aug - Jun | 5.15 | <0.001 |
| *A. nodosum* | Winter | Dec – Jan | -12.29 | <0.001 |
| *A. nodosum* | Winter | Jan - Feb | -16.84 | <0.001 |
| *A. nodosum* | Winter | Feb - Dec | 4.55 | <0.001 |
|  |  |  |  |  |
| *F. serratus* | Autumn | Sep - Oct | -2.33 | =0.449 |
| *F. serratus* | Autumn | Oct - Nov | -3.31 | =0.043 |
| *F. serratus* | Autumn | Nov - Sep | -5.65 | <0.001 |
| *F. serratus* | Spring | Mar - Apr | 1.13 | =0.993 |
| *F. serratus* | Spring | Mar - May | -2.75 | =0.201 |
| *F. serratus* | Spring | May - Apr | -1.62 | =0.902 |
| *F. serratus* | Summer | Jun - Jul | 2.39 | =0.408 |
| *F. serratus* | Summer | Jun - Aug | 3.84 | <0.01 |
| *F. serratus* | Summer | Aug - Jul | 1.45 | =0.953 |
| *F. serratus* | Winter | Dec – Jan | 0.25 | =1.000 |
| *F. serratus* | Winter | Jan - Feb | -1.15 | =0.992 |
| *F. serratus* | Winter | Feb - Dec | 1.41 | =0.960 |
|  |  |  |  |  |
| *F. vesiculosus* | Autumn | Sep - Oct | -1.97 | =0.711 |
| *F. vesiculosus* | Autumn | Oct - Nov | 2.03 | =0.668 |
| *F. vesiculosus* | Autumn | Nov - Sep | 0.06 | =1.000 |
| *F. vesiculosus* | Spring | Mar - Apr | -2.80 | =0.177 |
| *F. vesiculosus* | Spring | Mar - May | 6.31 | <0.001 |
| *F. vesiculosus* | Spring | Apr - May | 3.51 | =0.022 |
| *F. vesiculosus* | Summer | Jun - Jul | -7.68 | <0.001 |
| *F. vesiculosus* | Summer | Jul - Aug | 7.59 | <0.001 |
| *F. vesiculosus* | Summer | Aug - Jun | -0.08 | =1.000 |
| *F. vesiculosus* | Winter | Dec – Jan | 4.49 | <0.001 |
| *F. vesiculosus* | Winter | Jan - Feb | -0.34 | =1.000 |
| *F. vesiculosus* | Winter | Feb - Dec | 4.83 | <0.001 |
|  |  |  |  |  |
| *S. latissima* | Autumn | Sep - Oct | -10.30 | <0.001 |
| *S. latissima* | Autumn | Oct - Nov | -1.52 | =0.933 |
| *S. latissima* | Autumn | Nov - Sep | -11.83 | <0.001 |
| *S. latissima* | Spring | Mar - Apr | -0.44 | =1.000 |
| *S. latissima* | Spring | Mar - May | -7.21 | <0.001 |
| *S. latissima* | Spring | Apr - May | -7.66 | <0.001 |
| *S. latissima* | Summer | Jun - Jul | -3.27 | =0.048 |
| *S. latissima* | Summer | Jul - Aug | 0.60 | =1.000 |
| *S. latissima* | Summer | Aug - Jun | -2.67 | =0.236 |
| *S. latissima* | Winter | Dec – Jan | 6.94 | <0.001 |
| *S. latissima* | Winter | Jan - Feb | -1.92 | =0.744 |
| *S. latissima* | Winter | Feb - Dec | 8.86 | <0.001 |
